# Effect of Heat-Inactivated Compound Probiotics on Growth Performance, Plasma Biochemical Indices, and Cecal Microbiome in Yellow-Feathered Broilers

**DOI:** 10.3389/fmicb.2020.585623

**Published:** 2020-10-22

**Authors:** Cui Zhu, Li Gong, Kaiyong Huang, Fangjun Li, Diqing Tong, Huihua Zhang

**Affiliations:** School of Life Science and Engineering, Foshan University, Foshan, China

**Keywords:** probiotics, *Bacillus subtilis*, *Lactobacillus acidophilus*, broiler, microbiota, growth performance

## Abstract

This study was carried out to investigate the effect of heat-inactivated compound probiotics on growth performance, plasma biochemical indices, and gut microbiota composition and functions in yellow-feathered broilers. A total of 360 1-day-old broilers were randomly divided into 3 groups, including a basal diet as negative control group (PC), basal diet plus antibiotics with 250 mg/kg calcium oxytetracycline and 200 mg/kg Nosiheptide as positive control (PC), and basal diet plus 500 mg/kg compound probiotics consisting of heat-inactivated *Bacillus subtilis* and *Lactobacillus acidophilus* BFI (BFI). Each group had 6 replicates of 20 chickens. On d 21, 42, and 63, one chick from each replicate was selected for blood collection and cecal sampling. Compared to the NC group, dietary supplementation with heat-inactivated compound probiotics increased the feed efficiency during d 1–63 (*P* < 0.05). The plasma cholesterol content at 42 d and creatinine content at 63 d were decreased by dietary supplementation with heat-inactivated compound probiotics (*P* < 0.05). The dominant phyla in broiler cecal microbiota were *Bacteroidetes, Firmicutes*, and *Proteobacteria*, while the dominant genera were *Bacteroides, Ruminococcaceae*, and *Phascolarctobacterium*. The β-diversity index of cecal microbiota in BFI group was increased at d 42 (*P* < 0.01) and d 63 (*P* < 0.05). Dietary heat-inactivated compound probiotics increased the relative abundances of *Barnesiellaceae* (family), *Barnesiella* (genus), and *Lactobacillus aviarius* (species) at d 21, and reduced the relative abundances of genera *Lachnoclostridium* and *Peptococcus* at d 42, and unidentified *Lachnospiraceae* and *Lachnoclostridium* at d 63. The functional prediction of microbiota revealed that supplementation with heat-inactivated compound probiotics enriched the pathways related to methane metabolism, transcription machinery, purine metabolism and protein export. The Spearman's correlation analysis identified a significant correlation between cecal microbiota composition and overall feed efficiency and plasma metabolites. Collectively, dietary heat-inactivated compound probiotics with *Bacillus subtilis* and *Lactobacillus acidophilus* BFI enhanced feed efficiency, and decreased plasma cholesterol and creatinine contents, which might be associated with the modulation of community composition, diversity and functions of cecal microbiota in yellow-feathered broilers. These results indicated the potential of heat-inactivated probiotics used as alternatives to antibiotics for improvement of broiler health and productivity.

## Introduction

The sub-therapeutic doses of antibiotic growth promoters (AGPs) have been widely used in poultry production to prevent intestinal inflammation and improve growth rate and feed conversion efficiency for decades (Huyghebaert et al., 2011). However, due to the increasing spread of antibiotic resistances and potential hazards of drug residue contamination to human health and environment, thereby attempts to maximize the growth and health of commercial poultry by seeking natural alternatives to AGPs have been intensified (Hussein et al., 2020). Probiotics referring to the live strains of strictly selected microorganisms that confer health benefits on the host when administered in adequate amounts (Hill et al., 2014), have been applied as direct-fed microbial antibiotic alternatives to enhance growth, immunity, and gut health in poultry (Grant et al., 2018). The probiotic microorganisms mostly used in livestock nutrition included *Lactobacillus, Bacillus subtilis, Enterococcus faecium, Bifidobacterium*, and *Saccharomyces cerevisiae* (Markowiak and Śliżewska, 2018). Among these, *Lactobacillus* and *Bacillus* have been identified as the major probiotic bacteria in chicken microbiome, and have been already commercially available for use in poultry (Azad et al., 2018). Indeed, previous studies have shown that dietary *Lactobacillus acidophilus* exhibited positive effects on performance and gut health in chickens (Forte et al., 2018), while *Bacillus subtilis* strengthened intestinal barrier (Rhayat et al., 2019), enhanced the intestinal integrity and nutrients absorption (Latorre et al., 2015), and modulated the fecal microbiome to protected broiler chickens against *Salmonella* infection (Oh et al., 2017).

The microbiota resident in the gastrointestinal tract (GIT) plays an important role to the host including nutrient absorption, innate and adaptive immune stimulation, and pathogen invasion, thereby contributing to the enhancement of growth and well-being of chickens (Xiao et al., 2017). However, the dysbiosis of healthy microbiome would increase growth of pathogenic strains leading to low quality and contaminated poultry products (Sood et al., 2020). Many previous studies have confirmed that the gut microbiome can be shaped by dietary intervention strategies including the inclusion of probiotics (Pan and Yu, 2014; Groussin et al., 2017; Clavijo and Flórez, 2018). Moreover, the development of next-generation sequencing methods has dramatically advanced our knowledge about the beneficial potential of probiotic species or strains involving the modulation of composition structure, diversity, and function of the gut microbiota in commercial poultry (Azad et al., 2018).

Previous study has shown that both viable probiotic and inactivated probiotic positively affected growth performance and modulated the intestinal immune response in broilers (Palamidi et al., 2016) and in lay hens (Zhang et al., 2012). However, the weakness of live probiotics to remain viable under the process, production, and storage in feed industry has restrained their convenience of use in animal production. Therefore, there is an increasing interest in products based on non-viable microorganisms that could be relatively easy for standard manufacturing and extensive applications in practical production due to its longer shelf-life (Adams, 2010). Importantly, many non-viable microorganisms including heat-killed probiotic bacteria or their metabolites displayed favorable effects in modulation of immune responses, maintenance of intestinal barrier integrity, and health of animals (Chuang et al., 2007; Piqué et al., 2019). Furthermore, previous studies have identified the differences in microbiota between birds were associated with growth performance of broiler chickens (Stanley et al., 2012, 2013). However, it remained largely unknown concerning the effect of heat-killed probiotics on modulation of microbial community, as well as the interactions of microbiota composition with feed efficiency and plasma metabolites in yellow-feathered broilers. We therefore hypothesized that dietary heat-killed compound probiotics might regulate the composition and function of gut microbiota for better growth performance in yellow-feathered broilers.

Thus, this study was carried out to investigate the effect of dietary supplementation with a mixture of probiotic containing heat-inactivated *Lactobacillus acidophilus* and *Bacillus subtilis* on the growth performance, plasma biochemical indices, organ indexes, and cecal microbiome of yellow-feathered broilers.

## Materials and Methods

### Ethics Statement

The research protocol and all the experimental procedures were approved by the Animal Care and Use Committee of Foshan University.

### Animals, Diets, and Management

A total of 360 1-day-old healthy female yellow-feathered broilers with similar initial body weight (BW) (36.72 ±0.25 g) were selected and randomly allocated to one of three groups, including the negative control group (NC), the antibiotic group as positive control (PC), and the heat-inactivated compound probiotics group (BFI). There were 6 replicates per treatment group with 20 chicks each replicate. The broilers in the NC group were fed with a corn-soybean meal basal diet (Table 1) which were prepared to meet the nutrient requirements for yellow-feathered broilers recommended by the Ministry of Agriculture of the People's Republic of China (2004). The broilers in the PC group were fed with the basal diet supplemented with 250 mg/kg nazetide and 200 mg/kg oxytetracycline calcium. Moreover, the broilers in the BFI group were fed with the basal diet supplemented with 500 mg/kg compound probiotics. Furthermore, the preparation of compound probiotics consisted of heat-inactivated *Bacillus subtilis* (1 × 10^8^ cfu/g) and *Lactobacillus acidophilus BFI* (1 × 10^8^ cfu/g) at a ratio of 1:1, and was kindly provided by the Bioforte Biotechnology Co., Ltd. (Shenzhen, China). The heat-inactivated compound probiotics were treated under 80°C for 30 min and proved to be inactivated according to bacterium culture test before use.

**Table 1:** The ingredient and nutrient level of the basal diet.

**Item**	**Starter (1–21 d)**	**Grower (22–42 d)**	**Finisher (43–63 d)**
**Ingredient (%)**			
Corn	61.00	63.26	65.52
Soybean meal	32.00	28.00	24.00
Corn gluten meal	1.50	2.00	3.00
Soybean oil	1.40	2.50	3.50
Limestone	1.41	1.41	1.35
Dicalcium phosphate	1.33	1.33	1.33
DL-Met	0.18	0.15	0.12
L-Lys-HCl	0.18	0.18	0.18
Wheat middling	0.11	0.17	0.00
Vitamin-mineral premix^a^	1.00	1.00	1.00
**Calculated nutrient level**			
ME (MJ/kg)	12.12	12.54	12.96
CP (%)	19.91	18.63	17.60
Lys (%)	1.09	1.00	0.92
Met (%)	0.51	0.46	0.42
Ca (%)	0.87	0.88	0.84
Available *P* (%)	0.42	0.40	0.38

a *The premix provided the following per kg of diet: VA, 6,000 IU; VD_3_, 2,000 IU; VE, 30 mg; VK_3_, 2 mg; VB_1_, 3 mg; VB_2_, 5 mg; pantothenic acid, 800 mg; choline chloride, 1,500 mg; nicotinic acid, 30 mg; pyridoxine, 3 mg; folic acid, 500 mg; biotin, 0.2 mg; VB_12_, 1 mg; Fe, 100 mg; Cu, 8 mg; Mn, 100 mg; Zn, 100 mg; I, 0.42 mg; Se, 0.3 mg*.

The experimental period lasted 63 days, which included the starter (1–21 d), grower (22–42 d) and finisher (43–63 d) phases. During the starter phase, 20 broilers within each replicate were kept in a single cage (32.5 × 62 × 42 cm), while they were housed in 2 cages of this size with 10 broilers each during the grower and finisher phases. Feed and water were provided *ad libitum* to all broilers. The birds at 1-day old were vaccinated with standard procedure of Marek's disease vaccines for broilers. The ambient temperature was kept 35°C in the first week of life and maintained at 25°C thereafter, while maintaining a relative humidity of 60–70%. Light was provided for 22 h at the first week with a reduction to 16 h afterward.

### Growth Performance

On the d 21, 42, and 63 after 12-h feed withdrawal, the broilers in each replicate were weighed individually accompanied with the feed residues recorded, then the average daily gain (ADG), average daily feed intake (ADFI), and feed conversion ratio (FCR) were calculated accordingly after adjusting for the mortality during different phase (1–21 d, 22–42 d, and 43–63 d) and the whole experiment (1–63 d).

### Sampling

After weighing at d 21, 42, and 63, one broiler close to the average weight in each replicate was selected for harvesting blood samples followed by slaughter and tissue collections. Briefly, the blood samples (6 mL) were collected from the brachial vein, injected into a 10-mL anticoagulation tube containing EDTA, and then centrifugated at 1,320 × g, 4°C for 10 min to get the plasma. The plasma samples were separated into a 1.5 mL sterile eppendorf tube and labeled for storage at −80°C for further analysis of biochemical parameters. After blood collection, the whole intestine was quickly isolated from the abdominal cavity and placed on ice to separate the cecum section. Then, the cecum was washed with ice-old PBS followed by removal of the remaining PBS on the surface of cecum with filter paper. The cecal contents of each broiler were collected into a 5-mL sterile eppendorf tube, quickly frozen with liquid nitrogen, and then placed at −80°C for microbiome analysis by 16S rRNA amplicons sequencing. The microbial composition and diversity changes in cecal contents were analyzed to study the treatment effects. At d 63 of the experiment, the heart, spleen, liver, and bursa of Fabricius samples were collected and weighed individually to measure their organ indexes (ratio relative to final BW).

### Analyses of Plasma Biochemical Indicators

The plasma biochemical indicators including uric acid, creatinine, total protein (TP), albumin, cholesterol, triglyceride, and plasma enzymes alanine transaminase (ALT) and aspartate transaminase (AST) activities, were determined with standard commercial kits following the manufacturer's instructions using an Architect C8000 Automatic Biochemical Analyzer (Abbott, Inc., Chicago, IL, USA).

### DNA Extraction, 16S Sequencing, and Data Processing

Total DNA was extracted from cecal sample using a commercial kit according to the manufacturer's recommendations (Omega Bio-Tek, Norcross, USA). The DNA concentration was checked by NanoDrop 2000 Spectrophotometer (Thermo Fischer Scientific, Wilmington, USA), and the DNA quality was monitored by 1% agarose gel electrophoresis. The V3-V4 variable region of 16S rRNA gene was amplified using the specific primers (341 F: 5′-CCTAYGGGRBGCASCAG-3′; 806 R: 5′-GGACTACNNGGGTATCTAAT-3′). The PCR mixture with 30 μL reaction contained 15 μL of Phusion High-Fidelity PCR Master Mix (New England Biolab, Ipswich, USA), 1 μL of forward and reverse primers (0.2 μM), and 10 ng template DNA. The amplification profile consisted of initial denaturation at 98°C for 1 min, and 30 cycles of three steps (98°C for 10 s, annealing at 50°C for 30 s, and elongation at 72°C for 30 s), followed by a final elongation step at 72°C for 5 min. The PCR products were detected by agarose gel electrophoresis, and 300 bp amplicon was cleaned and subjected to 16S rDNA sequencing on an Illumina HiSeq 2500 PE 250 platform (Novogene Bioinformatics Technology Co., Ltd., Tianjin, China).

All sequence data processing was performed using the QIIME software package (Berg-Lyons et al., 2010). Sequences were paired-end and high-quality sequences were aligned against the SILVA database (Ribocon GmbH, Bremen, Germany) (Quast et al., 2013). The UCHIME software (Tiburon, CA, USA) was used to identify and remove chimeric sequences (Edgar et al., 2011). Operational taxonomic units (OTUs) were assigned at a 97% identity using the SILVA database. The Venn diagram with shared and unique OTUs was used to identify the similarity and difference among treatments. The clustered OTUs were used to calculate the alpha-diversity within groups including Shannon index, Simpson index, abundance based coverage estimators (ACE), Chao 1 richness, Good's coverage, and phylogenetic diversity (PD) of whole tree. Beta diversity index, principal coordinate analysis (PCoA plots, weighted UniFrac distance), Non-metric multidimensional scaling (NMDS plots, weighted UniFrac distance), and unweighted pair-group method with arithmetic means (UPGMA) clustering were accessed to calculate the β-diversity between groups. The differences in the relative abundances of microbiota among treatments were compared using the linear discriminant analysis effect size (LEfSe), *T*-test, and MetaStat analyses (Segata et al., 2011). To understand the potential functional profile of the cecal microbiota affected by dietary treatments, a Phylogenetic Investigation of Communities by Reconstruction of Unobserved States (PICRUSt) was carried out (Langille et al., 2013). The predicted Kyoto Encyclopedia of Genes and Genomes (KEGG) orthologs were summarized to Level-3 functional categories.

### Statistical Analysis

The data were subjected to statistical analysis by one-way ANOVA using the SPSS software (version 18.0) (Chicago, IL, USA). Significant differences between means were compared using Duncan's Multiple Range. The replicate (*n* = 6) was considered as the experimental unit. The correlations between cecal microbiota and feed efficiency together with the differential plasma biochemical indices were assessed by Spearman's correlation analysis. The results are presented as the mean ± standard error (SE). *P* < 0.05 was considered significantly different.

## Results

### Growth Performance

As shown in Table 2, compared to the NC group, dietary supplementation with heat-inactivated compound probiotics decreased the FCR of yellow-feathered broilers during d 1 to 63 (*P* < 0.05), indicating an improved feed efficiency through the overall period. However, there was no significant difference in initial BW, final BW, ADG, or ADFI at all phases among the three groups (*P* > 0.05).

**Table 2:** Effect of dietary supplementation with heat-inactivated compound probiotics on the growth performance in yellow-feathered broilers.

**Item**	**Treatment^1^**		
	**NC**	**PC**	**BFI**
Initial BW, g	36.56 ± 0.51	36.91 ± 0.31	36.69 ± 0.52
Final BW, g	1603.89 ± 41.31	1624.97 ± 7.20	1616.63 ± 19.21
**ADG, g/d**			
1–21 d	16.93 ± 0.59	17.23 ± 0.21	16.59 ± 0.11
22–42 d	31.86 ± 0.98	31.77 ± 0.27	30.79 ± 0.42
43–63 d	27.09 ± 1.11	27.19 ± 1.16	27.57 ± 1.13
1–63 d	25.68 ± 0.64	25.31 ± 0.80	25.33 ± 0.23
**ADFI, g/d**			
1–21 d	26.32 ± 0.25	26.60 ± 0.40	26.86 ± 0.28
22–42 d	69.03 ± 1.84	67.09 ± 0.37	64.77 ± 0.86
43–63 d	81.67 ± 2.11	77.33 ± 2.70	76.73 ± 0.29
1–63 d	58.97 ± 1.39	58.05 ± 0.41	56.21 ± 0.56
**FCR**			
1–21 d	1.56 ± 0.06	1.54 ± 0.01	1.62 ± 0.01
22–42 d	2.16 ± 0.02	2.11 ± 0.03	2.10 ± 0.03
43–63 d	3.02 ± 0.06	2.86 ± 0.16	2.79 ± 0.11
1–63 d	2.30 ± 0.01^a^	2.29 ± 0.02^a^	2.21 ± 0.02^b^

1 *NC, negative control without in-feed antibiotics; PC, positive control containing 200 mg/kg with oxytetracycline calcium + 250 mg/kg of nasiheptide; BFI, compound probiotics supplementation at 500 mg/kg containing heat-inactivated Bacillus subtilis and Lactobacillus acidophilus BFI at a ratio of 1:1*.

a,b *Means in the same row with different superscripts differ (P < 0.05)*.

### Plasma Biochemical Indicators

The plasma uric acid concentration was increased in PC group relative to that in NC group (*P* < 0.05), and there was no significant difference in uric acid between PC and BFI groups (*P* > 0.05; Table 3). Dietary supplementation with heat-inactivated compound probiotics decreased the plasma creatinine content at 63 d, compared to that of NC group and PC group (*P* < 0.05). The plasma cholesterol contents at 42 d in yellow-feathered broilers fed with heat-inactivated compound probiotics were significantly lower than that of the NC group, but higher at 63 d than that of PC group (*P* < 0.05). Dietary antibiotic supplementation increased the plasma ALT concentration at 21 and 63 d in yellow-feathered broilers when compared to the NC group (*P* < 0.05). Dietary heat-inactivated compound probiotics decreased the plasma ALT content at 63 d in comparison with the PC group (*P* < 0.05). However, there were no significant differences in plasma concentrations of total protein, albumin, triglyceride, and AST at all phases among the three groups (*P* > 0.05).

**Table 3:** Effect of dietary supplementation with heat-inactivated compound probiotics on the blood biochemical parameters in yellow-feathered broilers.

**Item**	**Treatment^1^**		
	**NC**	**PC**	**BFI**
**Uric acid (μmol/L)**			
21 d	181.50 ± 14.86^b^	269.67 ± 35.41^a^	256.50 ± 33.08^ab^
42 d	211.33 ± 19.00	226.17 ± 32.64	172.83 ± 12.29
63 d	257.50 ± 22.55	241.67 ± 25.35	299.00 ± 29.59
**Total protein (g/L)**			
21 d	32.87 ± 1.50	30.28 ± 0.72	30.87 ± 1.18
42 d	35.25 ± 0.81	34.95 ± 1.52	38.10 ± 3.62
63 d	37.45 ± 1.25	35.58 ± 0.99	37.03 ± 1.12
**Albumin (g/L)**			
21 d	13.88 ± 0.44	13.57 ± 0.79	13.20 ± 0.42
42 d	15.00 ± 0.74	14.42 ± 0.78	14.25 ± 0.28
63 d	14.95 ± 0.60	14.27 ± 0.30	15.32 ± 0.40
**Creatinine (μmol/L)**			
21 d	5.50 ± 0.50	6.00 ± 0.93	6.17 ± 1.01
42 d	5.17 ± 0.48	4.83 ± 0.54	4.17 ± 0.60
63 d	5.00 ± 0.73^a^	5.00 ± 0.00^a^	2.83 ± 0.54^b^
**Cholesterol (mmol/L)**			
21 d	3.53 ± 0.22	3.48 ± 0.24	3.47 ± 0.16
42 d	3.99 ± 0.23^a^	3.64 ± 0.22^ab^	3.25 ± 0.07^b^
63 d	3.56 ± 0.13^ab^	3.11 ± 0.15^b^	3.84 ± 0.24^a^
**Triglyceride (mmol/L)**			
21 d	0.45 ± 0.02	0.50 ± 0.04	0.47 ± 0.04
42 d	0.58 ± 0.05	0.53 ± 0.04	0.47 ± 0.01
63 d	0.52 ± 0.03	0.54 ± 0.02	0.52 ± 0.04
**ALT (U/L)**			
21 d	2.00 ± 0.37^b^	3.17 ± 0.48^a^	2.67 ± 0.21^ab^
42 d	2.00 ± 0.00	2.00 ± 0.26	2.00 ± 0.26
63 d	2.00 ± 0.26^b^	3.17 ± 0.40^a^	2.00 ± 0.45^b^
**AST(U/L)**			
21 d	201.50 ± 7.72	222.67 ± 7.13	211.83 ± 13.71
42 d	196.17 ± 11.95	218.17 ± 15.34	195.83 ± 3.55
63 d	214.00 ± 8.51	210.67 ± 17.69	230.33 ± 25.93

1 *NC, negative control without in-feed antibiotics; PC, positive control containing 200 mg/kg of oxytetracycline calcium + 250 mg/kg of nasiheptide; BFI, compound probiotics supplementation at 500 mg/kg containing heat-inactivated Bacillus subtilis and Lactobacillus acidophilus BFI at a ratio of 1:1; ALT, alanine transaminase; AST, aspartate transaminase*.

*Results are shown as mean ± standard error (n = 6)*.

a,b *Means in the same row with different superscripts differ (P < 0.05)*.

### Organ Indexes

The results of organ indexes of yellow-feathered broilers are shown in Table 4. However, there were no significant differences in the spleen, liver, heart, and bursa indexes of the yellow-feathered broilers at all phases among different groups (*P* > 0.05).

**Table 4:** Effect of dietary supplementation with heat-inactivated compound probiotics on the organ index in yellow-feathered broilers.

**Item**	**Treatment^a^**		
	**NC**	**PC**	**BFI**
**Bursa index**			
21 d	0.30 ± 0.05	0.25 ± 0.02	0.23 ± 0.04
42 d	0.18 ± 0.01	0.23 ± 0.04	0.26 ± 0.02
63 d	0.13 ± 0.02	0.14 ± 0.02	0.10 ± 0.02
**Spleen index**			
21 d	0.17 ± 0.02	0.18 ± 0.02	0.15 ± 0.01
42 d	0.17 ± 0.02	0.17 ± 0.01	0.19 ± 0.01
63 d	0.15 ± 0.01	0.20 ± 0.03	0.20 ± 0.03
**Liver index**			
21 d	3.23 ± 0.18	3.48 ± 0.15	3.82 ± 0.15
42 d	2.37 ± 0.11	2.16 ± 0.02	2.28 ± 0.10
63 d	1.72 ± 0.05	1.64 ± 0.07	1.79 ± 0.11
**Heart index**			
21 d	0.62 ± 0.05	0.63 ± 0.04	0.59 ± 0.03
42 d	0.43 ± 0.03	0.40 ± 0.02	0.48 ± 0.02
63 d	0.34 ± 0.01	0.39 ± 0.03	0.37 ± 0.02

a *NC, negative control without in-feed antibiotics; PC, positive control containing 200 mg/kg of oxytetracycline calcium + 250 mg/kg of nasiheptide; BFI, compound probiotics supplementation at 500 mg/kg containing heat-inactivated Bacillus subtilis and Lactobacillus acidophilus BFI at a ratio of 1:1*.

*Results are shown as mean ± standard error (n = 6)*.

### Cecal Bacterial Community Structure

**Figure 1:**
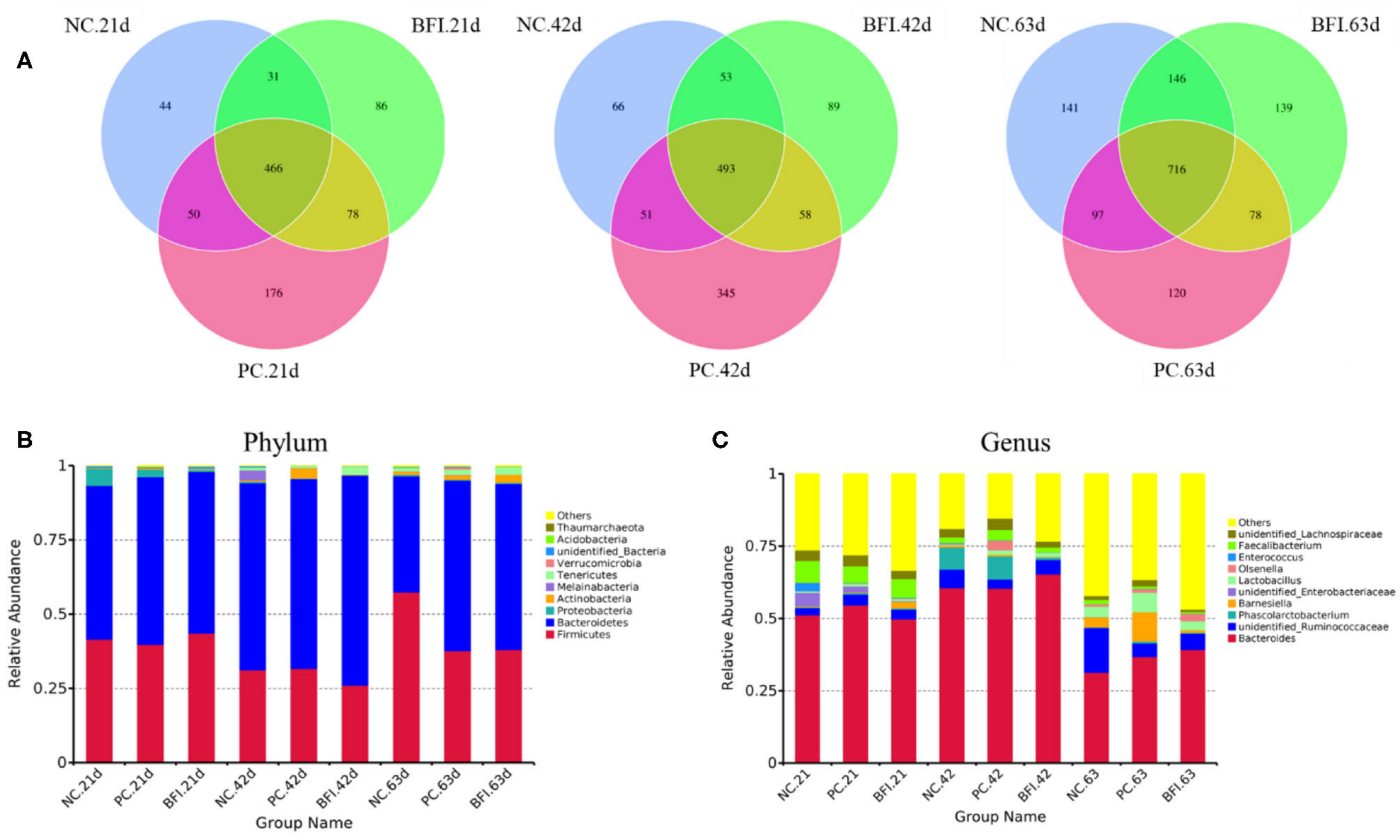
The Venn diagram and bacterial community composition of yellow-feathered broilers fed with antibiotics or compound probiotics-supplemented diets. **(A)** The Venn diagram of OTUs at starter, grower, and finisher phases. Microbial composition in the cecum at the phylum **(B)** or genus **(C)** level. Relative abundance <1% were combined into “other.” NC, negative control without in-feed antibiotics. PC, positive control with in-feed antibiotics with 200 mg/kg of oxytetracycline calcium + 250 mg/kg of nasiheptide. BFI, compound probiotics consisting of heat-inactivated *Bacillus subtilis* and *Lactobacillus acidophilus* BFI.

The Illumina HiSeq 250 pyrosequencing of the V3-V4 region of 16S rRNA genes were used to amplify the bacterial DNA, and thus to quantitatively characterize the cecal microbial changes in yellow-feathered broilers. The Venn diagram (Figure 1A) showed that the cecal microbiota of yellow-feathered broilers at d 21 shared 466 OTUs across the three groups, and the unique OUTs were 44, 179, and 86 in the NC, PC, and BFI group, respectively. At d 42, there were 493 common OTUs among the three groups, while unique OUT numbered 345 in NC group, 66 in PC group, and 89 in BFI group. At d 63, common OTUs within the three groups were 716, with unique 141, 139, and 120 OTUs in the NC, PC, and BFI, respectively.

The composition of top 10 phyla (Figure 1B) and genera (Figure 1C) in cecal contents of yellow-feathered broilers are shown. The dominant phyla of cecal microbiota were *Bacteroidetes, Firmicutes, Proteobacteria, Actinobacteria, Melainabacteria, Tenericutes, Verrucomicrobia*, unidentified *Bacteria, Acidobacteria*, and *Thaumarchaeota* (Figure 1B). At the genus level, the dominant microbiota in cecal contents were *Bacteroides*, unidentified *Ruminococcaceae, Phascolarctobacterium, Barnesiella*, unidentified *Enterobacteriaceae, Lactobacillus, Olsenella, Enterococcus, Faecalibacterium*, and unidentified *Lachnospiraceae* (Figure 1C).

### Microbial Diversity of the Broiler Cecal Contents

The α-diversity (Table 5) of the cecal microbiota in yellow-feathered broilers at different phases are provided. Nevertheless, there were no significant effects of antibiotic or heat-inactivated compound probiotics treatments in these parameters of α-diversity, including observed species, Chao 1, Shannon index, Simpson index, Goods coverage, ACE, and PD whole tree (*P* > 0.05).

**Table 5:** Effect of dietary supplementation with heat-inactivated compound probiotics on the α-diversity in yellow-feathered broilers.

**Item**	**Treatment^a^**		
	**NC**	**PC**	**BFI**
**Observed species**			
21 d	301.80 ± 16.54	318.80 ± 34.77	316.80 ± 7.57
42 d	319.50 ± 31.02	333.60 ± 6.39	300.20 ± 32.34
63 d	471.40 ± 28.42	472.60 ± 29.13	460.20 ± 62.87
**Chao1**			
21 d	304.06 ± 16.70	320.99 ± 16.70	319.27 ± 7.65
42 d	324.02 ± 31.74	337.17 ± 6.36	304.19 ± 33.43
63 d	476.48 ± 27.63	477.12 ± 29.25	465.75 ± 63.85
**Shannon index**			
21 d	3.98 ± 0.30	3.63 ± 0.40	4.51 ± 0.43
42 d	4.04 ± 0.25	4.04 ± 0.12	3.89 ± 0.32
63 d	4.67 ± 0.36	4.66 ± 0.26	4.80 ± 0.32
**Simpson index**			
21 d	0.80 ± 0.02	0.68 ± 0.07	0.83 ± 0.05
42 d	0.87 ± 0.02	0.84 ± 0.02	0.80 ± 0.07
63 d	0.86 ± 0.04	0.89 ± 0.01	0.90 ± 0.02
**Goods coverage**			
21 d	0.99965 ± 0.00002	0.99965 ± 0.00009	0.99963 ± 0.00002
42 d	0.99947 ± 0.00007	0.99953 ± 0.00004	0.99949 ± 0.00001
63 d	0.99932 ± 0.00004	0.99936 ± 0.00007	0.99927 ± 0.00007
**Abundance based coverage estimators (ACE)**			
21 d	309.41 ± 16.88	326.48 ± 36.96	325.07 ± 7.62
42 d	331.92 ± 32.14	344.32 ± 6.69	313.61 ± 35.14
63 d	486.44 ± 27.81	486.52 ± 29.98	477.06 ± 27.81
**PD whole tree**			
21 d	21.59 ± 1.51	22.10 ± 2.97	20.45 ± 0.45
42 d	19.94 ± 1.32	21.45 ± 1.27	20.29 ± 2.46
63 d	32.86 ± 2.77	34.50 ± 2.67	35.12 ± 3.71

a *NC, negative control without in-feed antibiotics. PC, positive control with in-feed antibiotics 200 mg/kg of oxytetracycline calcium + 250 mg/kg of nasiheptide. BFI, compound probiotics consisting of heat-inactivated Bacillus subtilis and Lactobacillus acidophilus BFI*.

**Figure 2:**
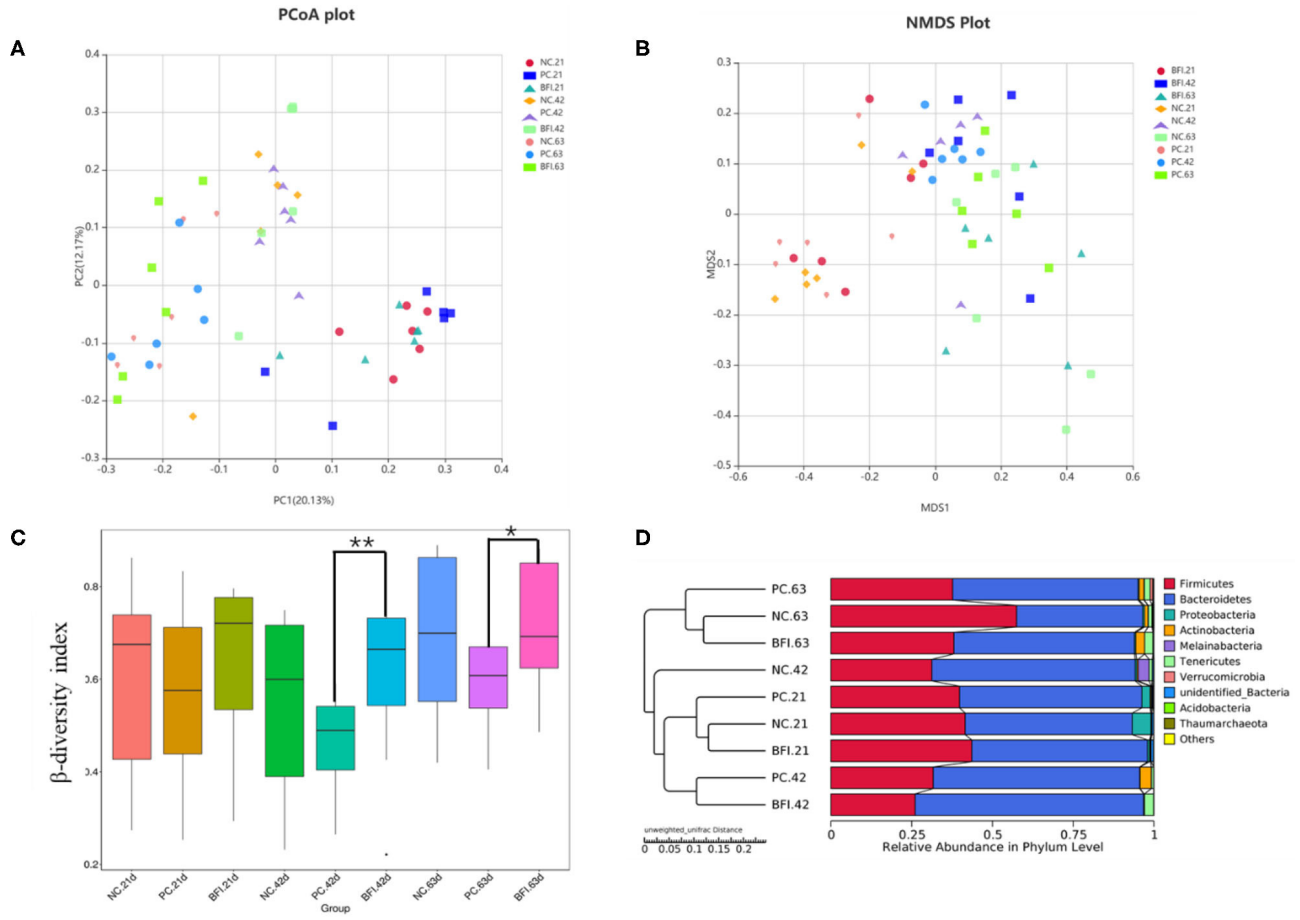
Effect of dietary compound probiotics on the β-diversity of cecel microbiota in yellow-feathered broilers. **(A)** PCoA plot. **(B)** NMDS plot. **(C)** β-diversity index. **(D)** UPGMA clustering was conducted based on unweighted unifrac distance. NC, negative control without in-feed antibiotics. PC, positive control with in-feed antibiotics with 200 mg/kg of oxytetracycline calcium + 250 mg/kg of nasiheptide. BFI, compound probiotics consisting of heat-inactivated *Bacillus subtilis* and *Lactobacillus acidophilus* BFI. ^*^P < 0.05 and ^**^P < 0.01.

The β-diversity of the cecal microbiota in yellow-feathered broilers at different phases are shown in Figure 2. The results of PCoA plot (Figure 2A) and NMDS plot (Figure 2B) suggested that microbial community in cecum formed a distinct cluster and separated from each group and phase. Based on Bray-Curtis dissimilarity, the β-diversity index of cecal microbiota in BFI group at d 42 (*P* < 0.01) and d 63 (*P* < 0.05) was significantly higher than that of PC group at the same timepoints (Figure 2C). The UPGMA clustering based on unweighted unifrac distance also confirmed the distinct differences in the microbiota composition of cecal contents from 3 groups and 3 ages (Figure 2D). Especially for the NC group at d 42, it was distinctly separated from other groups at different ages as a single cluster (Figure 2D).

### LEfSe Analysis of Broiler Cecal Microbiota

**Figure 3:**
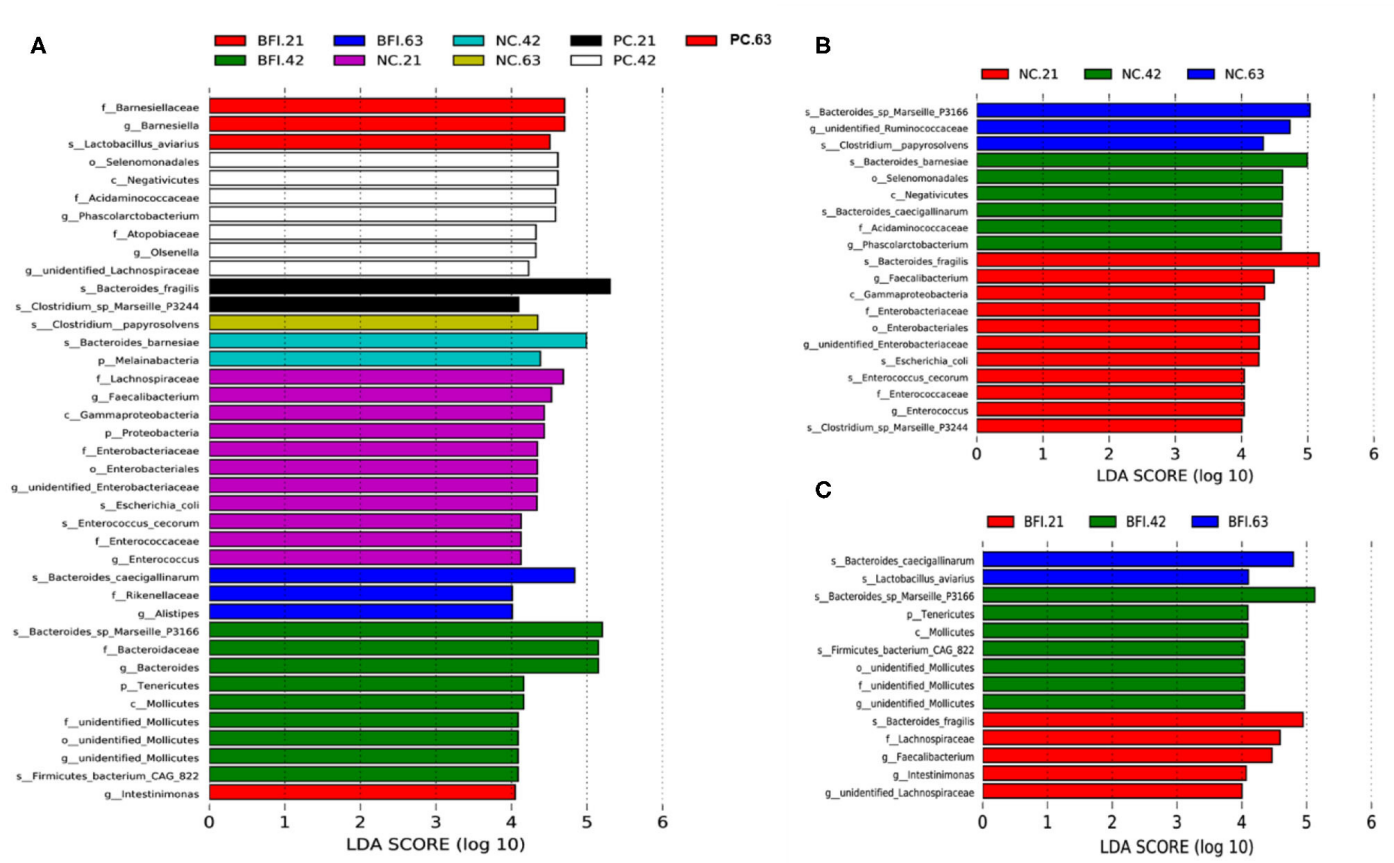
The LEfSe analysis of cecal microbiota in yellow-feathered broilers fed with antibiotic or compound probiotic-supplemented diets. The LEfSe analysis of cecal microbiota among treatment groups at different phases **(A)**. The LEfSe analysis of cecal microbiota in NC group **(B)** or BFI group **(C)**. NC, negative control without in-feed antibiotics. PC, positive control with in-feed antibiotics with 200 mg/kg of oxytetracycline calcium + 250 mg/kg of nasiheptide. BFI, compound probiotics consisting of heat-inactivated *Bacillus subtilis* and *Lactobacillus acidophilus* BFI.

The differential microbiota from different treatments are presented based on LEfSe analysis (Figure 3A), it showed that *Barnesiellaceae* (family), *Barnesiella* (genus), and *Lactobacillus aviarius* (species) were enriched in BFI group at d 21. There were 9 bacterial taxa enriched in BFI group at d 42, which consisted of *Bacteroides* sp. *Marseille P3166* (species), *Bacteroidaceae* (family), *Bacteroides* (genus), *Tenericutes* (phylum), *Molicutes* (class), unidentified *Mollicutes* (family, order, genus), and *Firmicutes bacterium CAG 822* (species). Three bacterial taxa including *Bacteroides caecigalinarum* (species), *Rikenellaceae* (family), and *Alistipes* (genus) were enriched in BFI group at d 63 (Figure 3A). Moreover, at d 21, a total of 11 bacterial taxa were enriched in NC group, which included *Lachnospiraceae* (family), *Faecalibacterium* (genus), *Gammaproteobacteria* (class), *Proteobacteria* (phylum), *Enterobacteriaceae* (family), *Enterobacteriales* (order), unidentified *Enterobacteriaceae* (genus), *Escherichia coli* (species), *Enterococcus cecorum* (species), *Enterococcaceae* (family), and *Enterococcus* (genus). At d 42, NC group enriched 2 bacterial taxa of *Bacteroides barnesiae* (species) and *Melainabacteria* (phylum), while only *Clostridium papyrosolvens* (species) was enriched in NC group at d 63 (Figure 3A). In addition, there was only 2 bacterial taxa of *Bacteroides fragilis* (species) and *Clostridium* sp. *Marseille P3244* enriched in PC group at d 21, while 7 bacterial taxa were enriched in PC group at 42, including *Selenomonadales* (order), *Negativicutes* (class), *Acidaminococcaceae* (family), *Phascolarctobacterium* (genus), *Atopobiaceae* (family), *Olsenella* (genus), and unidentified *Lachnospiraceae* (genus). Furthermore, only 1 bacterial taxum (*Intestinimonas*) at the genus level was enriched in PC group at d 63 (Figure 3A).

The LEfSe analysis of differential microbiota in NC group (Figure 3B) and BFI group (Figure 3C) from different phases (21, 42, and 63 d) are also presented. Totally, 11 bacterial taxa, such as *Bacteroides fragilis* (species), *Faecalibacterium* (genus), *Enterobacteriaceae* (family), *Enterobacteriales* (order), unidentified *Enterobacteriaceae* (genus), *Escherichia coli* (species), *Enterococcus cecorum* (species), *Enterococcaceae* (family), *Enterococcus* (genus), and *Clostridium* sp. *Marseille P3244*, were enriched in the NC group at d 21 (Figure 3B). There were 6 bacterial taxa enriched in NC group at 42 d, including *Bacteroides bamesiae* (species), *Selenomonadales* (order), *Negativicutes* (class), *Bacteroides caecigallinarum* (species), *Acidaminococcaceae* (family), *Phascolarctobacterium* (genus) (Figure 3B). The NC group at d 63 enriched *Bacteroides* sp. *Marseille P3166* (species), unidentified *Ruminococcaceae* (genus), and *Clostridium papyrosolvens* (species) compared to d 21 and d 42 (Figure 3B). Furthermore, the BFI group at d 21 enriched *Bacteroides fragilis* (species), *Lachnospiraceae* (family), *Faecalibacterium* (genus), *Intestinimonas* (genus), and unidentified *Lachnospiraceae* (genus) (Figure 3C). There were 7 bacterial taxa enriched in BFI group at d 42, including *Bacteroides* sp. *Marseille P3166* (species), *Tenericutes* (phylum), *Mollicutes* (class), *Firmicutes bacterium CAG 822* (species), and unidentified *Mollicutes* at order, family, and genus levels (Figure 3C). Compared to d 21 and 42, the BFI group at d 63 enriched the *Bacteroides caecigallinarum* and *Lactobacillus aviarius* at the species level (Figure 3C).

### T-test of Differential Microbiota in Cecal Contents of Broilers

**Figure 4:**
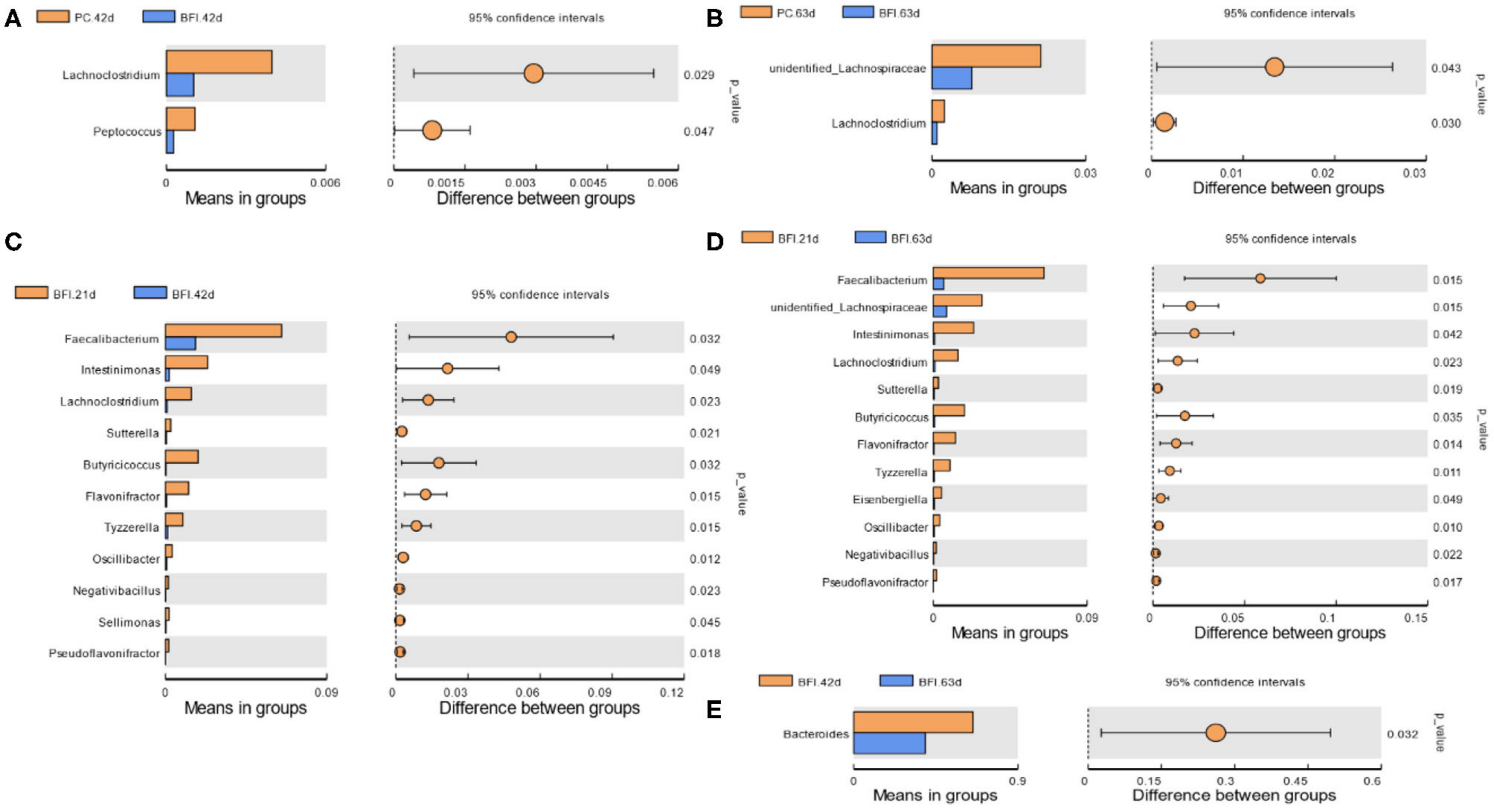
T-test analysis for the significant changes of differential microbiota at genus level in the cecal contents of yellow-feathered broilers. NC, negative control without in-feed antibiotics. PC, positive control with in-feed antibiotics with 200 mg/kg of oxytetracycline calcium + 250 mg/kg of nasiheptide. BFI, compound probiotics consisting of heat-inactivated *Bacillus subtilis* and *Lactobacillus acidophilus* BFI. **(A)** PC. 42 d vs. BFI 42 d. **(B)** PC. 63 d vs. BFI 63 d. **(C)** BFI. 21 d vs. BFI 42 d. **(D)** BFI. 21 d vs. BFI 63 d. **(E)** BFI. 42 d vs. BFI 63 d.

Compared to the PC group at d 42 or d 63, dietary supplementation with heat-inactivated compound probiotics (BFI group) reduced the relative abundances of *Lachnoclostridium* and *Peptococcus* at d 42 (Figure 4A) as well as unidentified *Lachnospiraceae* and *Lachnoclostridium* at d 63 (Figure 4B). The relative abundances of *Faecalibacterium, Intestinimonas, Lachnoclostridium, Sutterella, Butyricicoccus, Flavonifractor, Tyzzerella, Oscillibacter, Negativibacillus, Sellimonas*, and *Pseudoflavonifracto*r were higher in BFI group at 21 d than those at d 42 (Figure 4C). Moreover, the relative abundances of *Faecalibacterium*, unidentified *Lachnospiraceae, Intestinimonas, Lachnoclostridium, Sutterella, Butyricicoccus, Flavonifractor, Tyzzerella, Eisenbergiella, Oscillibacter, Negativibacillus*, and *Pseudoflavonifractor* were also increased at d 21 compared to those at d 63 (Figure 4D). The relative abundance of *Bacteroides* in BFI group at d 42 were higher than that at d 63 (Figure 4E).

### MetaStat Analysis of Cecal Microbiota Changes in Broilers

**Figure 5:**
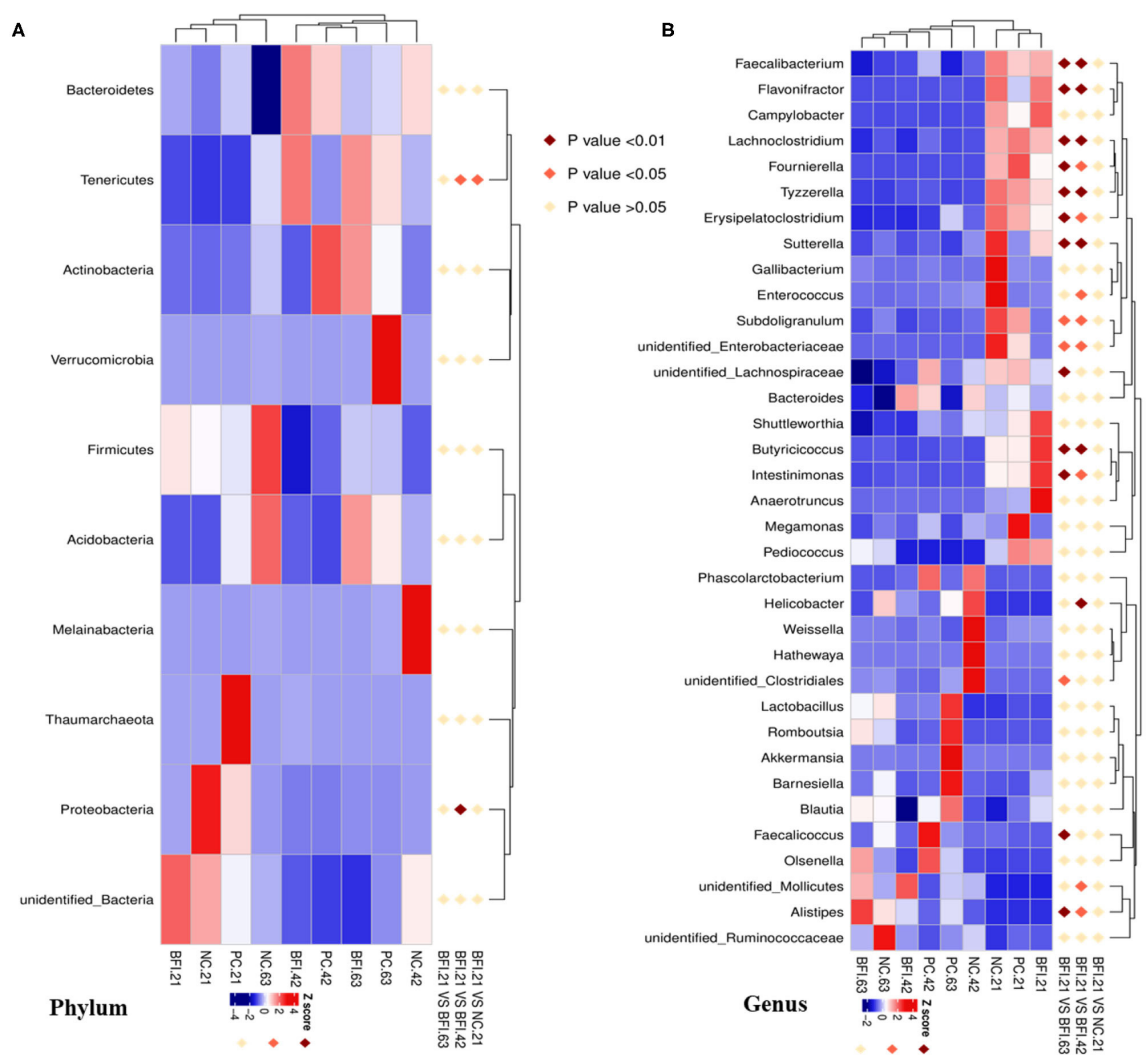
The Metastat analysis of cecal microbiota changes in yellow-feathered broilers fed with heat-inactivated compound probiotics. The MetaStat analysis revealed the dynamic changes of differential bacterial at the phylum **(A)** and genus level **(B)** in BFI group when fed with heat-inactivated compound probiotics for different periods. Z score marked in red and blue representing significant increase and decrease of the relative abundance of specific bacterial, respectively. NC, negative control without in-feed antibiotics. PC, positive control with in-feed antibiotics with 200 mg/kg of oxytetracycline calcium + 250 mg/kg of nasiheptide. BFI, compound probiotics consisting of heat-inactivated *Bacillus subtilis* and *Lactobacillus acidophilus* BFI.

Interestingly, the MetaStat analysis revealed the dynamic changes of differential bacterial at the phylum (Figure 5A) and genus level (Figure 5B) in BFI group when fed with heat-inactivated compound probiotics for different periods (21, 42, or 63 d). The relative abundances of *Tenericutes* and *Proteobacteria* at the phylum level (Figure 5A) were increased by heat-inactivated compound probiotics treatment for 21d compared to that for 42 d (BFI 21 vs. BFI. 42). Furthermore, at the genus level, the relative abundances of *Faecalibacterium, Flavonifractor, Lachnoclostridium, Fournierella, Tyzzerella, Erysipelatoclostridium, Sutterella, Subdoligranulum*, unidentified *Enterobacteriaceae, Butyricicoccus*, and *Intestinimonas* were reduced at d 42 or 63 d, while the relative of *Alistipes* was decreased in BFI group at d 21 (BFI 21 vs. BFI. 42 or BFI 21 vs. BFI. 63). These results suggested that the potential pathogenic bacteria might be reduced due to the extended treatment with heat-inactivated compound probiotics in yellow-feathered broilers.

### Predicted Microbial Function Analysis by PICRUSt

**Figure 6:**
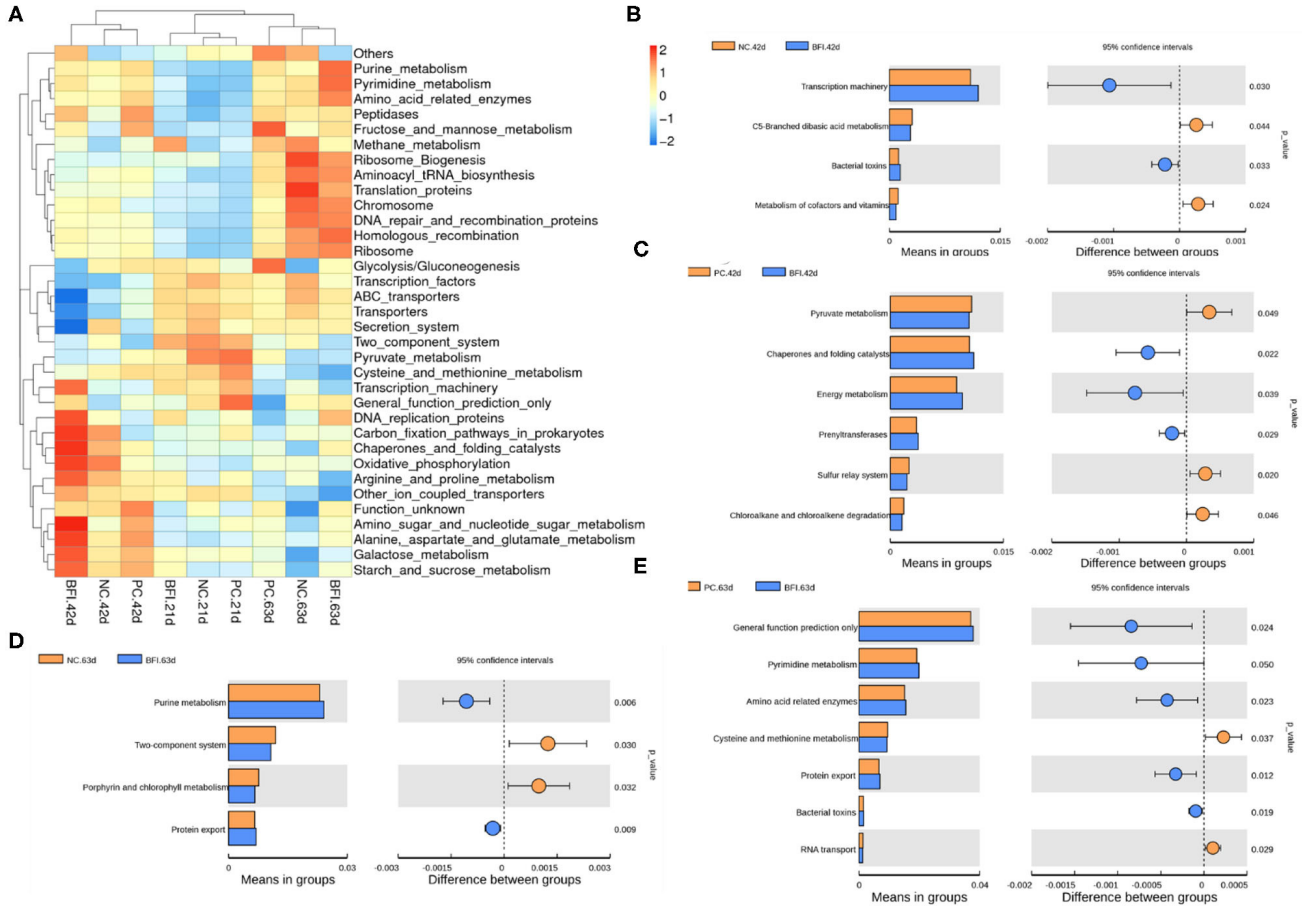
Predicted microbial function of cecal microbiota in yellow-feathered broilers. **(A)** The heatmap of microbial functions predicted by PICRUSt. **(B–E)** The comparisons of cecal microbial functions between treatments at different ages. Statistics were conducted by two-sided Welch's *t*-test and Benjamini-Hochberg FDR correction between pairs of means, with *P*-value lower than 0.05 indicating a significant difference in microbial function at Level 3 of KEGG pathways. NC, negative control without in-feed antibiotics. PC, positive control with in-feed antibiotics with 200 mg/kg of oxytetracycline calcium + 250 mg/kg of nasiheptide. BFI, compound probiotics consisting of heat-inactivated *Bacillus subtilis* and *Lactobacillus acidophilus* BFI.

The KEGG hierarchically clustered heat map analysis (Level 3, Figure 6A) showed that the pathway of methane metabolism was enriched in the BFI group at d 21. The functional pathways associated with transcription machinery, DNA replication protein, carbon fixation pathways in prokaryotes, chaperones, and folding catalysts, oxidative phosphorylation, arginine and proline metabolism, amino sugar and nucleotide glutamate metabolism, galactose metabolism, and starch and sucrose metabolism, were enriched in the BFI group at d 42. The pathways belonged to purine metabolism, pyrimidine metabolism, amino acid related enzymes, ribosome biogenesis, aminoacyl tRNA biosynthesis, translation proteins, chromosome, DNA repair and recombination proteins, homologous recombination, and ribosome were enriched in BFI group at d 63.

To identify the comparison of predicted functional pathways in cecal microbiota between treatments, two-sided Welch's *t*-test and Benjamini-Hochberg FDR correction between pairs of means were conducted (Figures 6B–E). Dietary supplementation with heat-inactivated compound probiotics for 42 d enriched the pathways of transcription machinery and bacterial toxins, but depressed those related to C5-branched dibasis acid metabolism as well as metabolism of cofactors and vitamins, when compared to the NC group at 42 d (Figure 6B). The pathways involved in chaperones and folding catalysts, energy metabolism, and prenyltransferases were enriched in BFI group at d 42, while those associated with pyruvate metabolism, sulfur relay system, chloroalkane, and chloroalkene degradation were depressed in BFI group at d 42 in relative to PC group at this time (Figure 6C). At d 63, dietary supplementation with heat-inactivated compound probiotics enriched the pathways belonging to purine metabolism and protein export, but depressed those involved in two-component system and porphyrin and chlorophyll metabolism, in comparison with NC group (Figure 6D). Moreover, when compared to the PC group at d 63, the pathways related to general function prediction, pyrimidine metabolism, amino acid related enzymes, protein export, and bacterial toxins were enriched in BFI group at 63, while those of cysteine and methionine metabolism as well as RNA transport were depressed in BFI group at 63 (Figure 6E).

### Spearman Correlation Analysis Between Cecal Microbiome and Feed Efficiency and Plasma Metabolites

**Figure 7:**
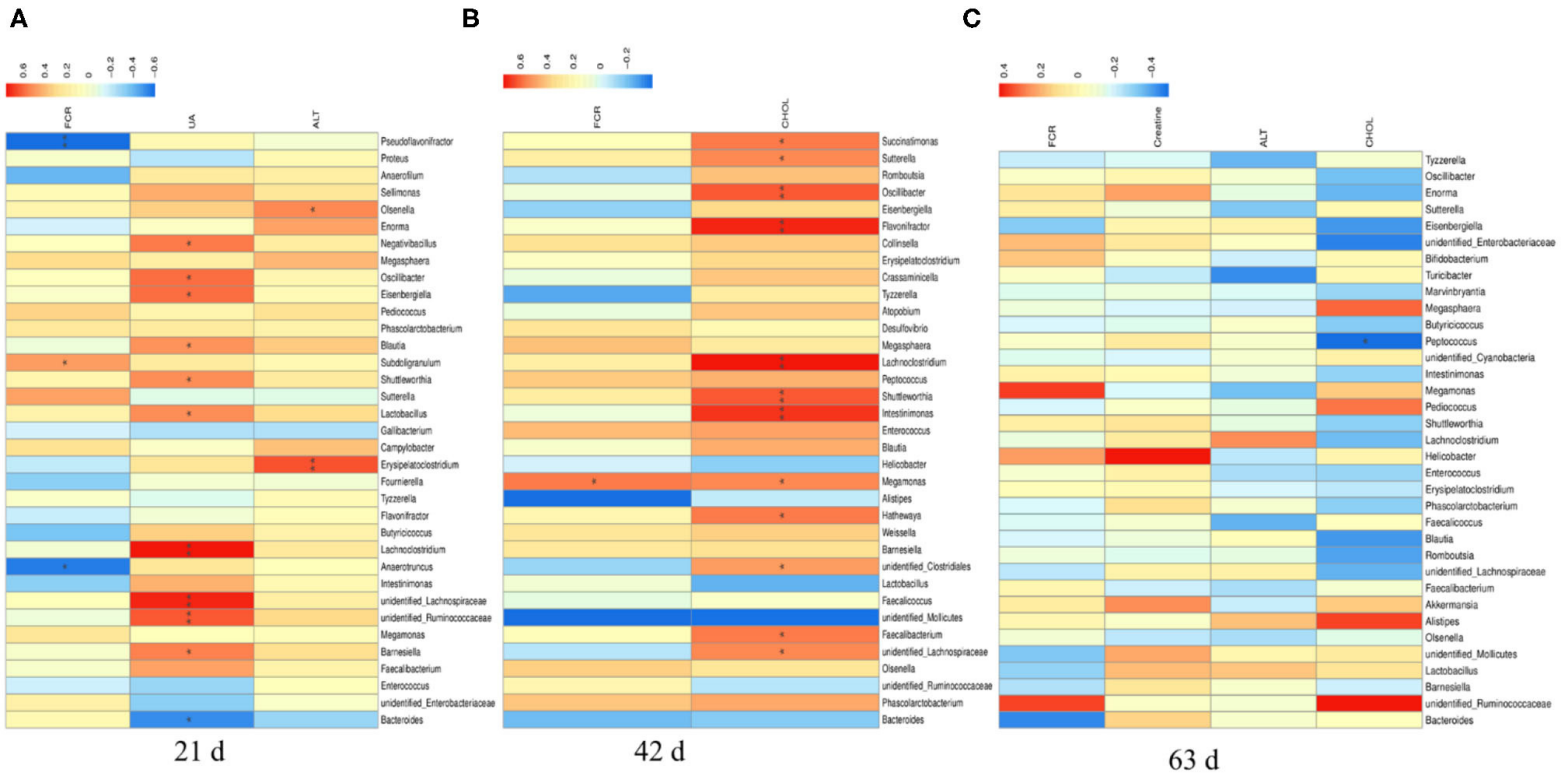
The Spearman correlation analysis of cecal microbial species at genus level with feed efficiency and plasma differential metabolites in yellow-feathered broilers. The overall feed efficiency, indicated by feed conversion ratio (FCR) at d 1–63, as well as the differential metabolites in the plasma at either d 21 **(A)**, 42 **(B)**, or 63 **(C)** were used to correlate the changes of cecal microbial species at genus level determined at corresponding phases. The heatmap with red indicated a positive correlation, while blue represented a negative correlation. UA, uric acid; ALT, alanine transaminase; CHOL, cholesterol. ^*^P < 0.05 and ^**^P < 0.01.

There were significant correlations between the top 35 cecal microbiota at genus level with overall FCR and plasma differential metabolites in yellow-feathered broilers (Figure 7). At d 21 (Figure 7A), the overall FCR (at 1–63 d) were negatively associated with the relative abundances of *Pseudoflavonifractor* (*P* < 0.01) and *Anaerotruncus* (*P* < 0.05), but positively associated with the relative abundance of *Subdoligranulum* (*P* < 0.05). The plasma uric acid at d 21 was positively correlated to the relative abundances of *Negativibacillus, Oscillibacter, Eisenbergiella, Blautia, Shuttleworthia, Lactobacillus*, and *Barnesiella* (*P* < 0.05), and *Lachnoclostridium*, unidentified *Lachnospiraceae*, unidentified *Ruminococcaceae* (*P* < 0.01), but negatively associated with that of *Bacteroides* at this time (*P* < 0.05). Furthermore, the relative abundances of *Olsenella* (*P* < 0.05) and *Erysipelatoclostridium* (*P* < 0.01) at d 21 were positively associated with the plasma ALT concentration at d 21.

At d 42 (Figure 7B), the overall FCR were positively associated with the relative abundance of *Megamonas* (*P* < 0.05). The plasma cholesterol concentration at d 42 was positively correlated to the relative abundances of *Succinatimonas, Sutterella, Megamonas, Hathewaya*, unidentified *Clostridiales, Faecalibacterium*, unidentified *Lachnospiraceae* (*P* < 0.05), and *Oscillibacter, Flavonifractor, Lachnoclostridium, Shuttleworthia*, and *Intestinimonas* at this time (*P* < 0.01). Moreover, there was a negative correlation between the relative abundance of *Peptococcus* at d 63 and the plasma concentration of cholesterol at this time (*P* < 0.05; Figure 7C).

## Discussion

Chickens are the most efficient agricultural species in converting feed to lean meat, and the quantity and quality of chicken meat can be improved by dietary nutrition and antibiotic applications. However, the increasing concern of antibiotic resistance and drug residues in egg and meat products by antibiotic abuse makes many countries ban the manufacturing, marketing and use of in-feed antibiotics, and thus the use of probiotics as safe alternatives in poultry diets is rapidly expanding (Huyghebaert et al., 2011).

Accumulating evidence has demonstrated the comparable health-beneficial effects of live probiotics to AGPs in terms of their efficiency to enhance poultry growth, inhibit pathogens and improve systemic and intestinal immunity (Jayaraman et al., 2017; Al-Khalaifah, 2018). For example, dietary *Bacillus subtilis* addition at 200 mg/kg significantly has been shown to improve the growth performance of broilers (Gao et al., 2017), and *Bacillus subtilis* supplementation displayed comparable results to antibiotics based on the results of feed intake, body weight gain and FCR in broiler infected with *Salmonella* (Abudabos et al., 2019). The *Bacillus*-based probiotics are especially popular due to their spore-forming properties and can be easy to handle in processed feed (Rhayat et al., 2017). Moreover, *Lactobacillus acidophilus* supplementation significantly enhanced the body weight gain and improved FCR in broilers under normal condition (De Cesare et al., 2017), and increased the BW on d 21 in broilers challenged with *Clostridium perfringens* (Li et al., 2018). When administered the mixture of *Lactobacillus* strains at 0.1%, the final BW, ADG and ADFI of broilers could be effectively attenuated under the higher temperature (35°C) compared to the lower temperature environment (24°C) (Faseleh Jahromi et al., 2016). In addition, previous study has demonstrated that multi-strain probiotics supplementation containing *Lactobacillus acidophilus, Bacillus subtilis*, and *Clostridium butyricum* in broilers improved growth performance, ileal amino acids digestibility and humoral immunity, and decreased the cecal numbers of *Escherichia coli* (Zhang and Kim, 2014). However, these aforementioned studies mainly focused on the use of live microorganisms in poultry production. Considering the disadvantages of live probiotics during manufacturing, storage, and usage, the non-viable probiotics together with their metabolites may represent an important alternative approach in modulation of poultry health (Adams, 2010; Piqué et al., 2019).

In the present study, we found that the overall feed efficiency was improved by dietary heat-inactivated compound probiotics mixture with *Bacillus subtilis* and *Lactobacillus acidophilus* BFI indicating more efficient of feed consumption to achieve better performance (Clavijo and Flórez, 2018). Our results were in accordance with previous results that the combination of a plant extract *Alisma canaliculatum* with probiotics containing *Lactobacillus acidophilus, Enterococcus faecium, Bacillus subtilis*, and *Saccharomyces cerevisiae* has been shown to improve the FCR in broilers (Hossain et al., 2012). Since it remains quite difficult for any single feed additive to completely replace the antibiotics, our results suggest that heat-inactivated compound probiotics might have great applications in animal husbandry when the importance of nutrition, feed processing, management, biosafety strategies is highly recognized during the antibiotic-free era.

Uric acid is the main end product in protein metabolism of birds, with high levels in blood related to kidney or liver problems. In the present study, the uric acid content in plasma were significantly increased by dietary antibiotic supplementation. Although *Bacillus subtilis* has been shown to increase the serum albumin in broilers exposed to *Salmonella* (Abudabos et al., 2017), here we found that dietary supplementation with heat-inactivated compound probiotics did not affect the metabolites of protein metabolism such as uric acid, total protein, and albumin. However, the creatinine as the metabolic product of creatine in muscle was significantly reduced by heat-inactivated compound probiotics. Higher concentrations of AST and ALT in blood are considered as major indicators of liver damage. Previous study has demonstrated that the serum AST and ALT concentrations in broilers were not affected by dietary *Bacillus subtilis* treatment for either 1 week or 2 weeks (Abudabos et al., 2017). Here, we showed that treatment with heat-inactivated compound probiotics for 63 d might display positive effect on liver health reflected by the decreased plasma concentration of ALT. Moreover, dietary heat-inactivated *Lactobacillus* and *Bacillus subtilis* supplementation could reduce the serum triglyceride and LDL cholesterol concentrations instead of serum total cholesterol in laying hens (Zhang et al., 2012). On the contrary, the plasma cholesterol at the grower phase was decreased by heat-inactivated compound probiotics in yellow-feathered broilers. Our results were consistent with previous results conducted in boilers (Mohan et al., 1996) and in laying hens (Kurtoglu et al., 2004) that dietary probiotic supplementation induced significant decrease of serum cholesterol concentration. The reduction of cholesterol by heat-killed *Lactobacilli* cells might be probably attributed to its abilities in assimilation of cholesterol during growth of *Lactobacilli*, incorporation of cholesterol into the cell membrane of *Lactobacilli*, and binding of cholesterol to the cell surface of *Lactobacilli* (Liong and Shah, 2005). These results suggested that compound probiotics, supplied in heat-activated form, might modulate the body metabolism of yellow-feathered broilers, of which the potential mechanism needs further investigations.

Gut microbiome plays an important role in maintaining the gut health, normal physiological functions, and productivity of poultry. Understanding the potential interaction between gut microbiome of poultry and the host and diet will help develop nutrition interventions for optimal health, growth and productivity in poultry (Pan and Yu, 2014). The cecum is the ideal habitat for diverse and complex microbiota, and harbors the highest bacterial density along the entire chicken gut. High-throughput sequencing has been commonly used to investigate the changes in the composition, diversity, and function of the gut microbiota by probiotics. In the present study, we used the 16S rRNA gene amplicons sequencing to investigate the composition and function of cecal contents in yellow-feathered broilers. We found that the primary phyla were *Bacteroidetes, Firmicutes, Proteobacteria, Actinobacteria, Melainabacteria*, and *Tenericutes*, while the dominant genera were *Bacteroides, Ruminococcaceae, Phascolarctobacterium, Barnesiella, Enterobacteriaceae*, and *Lactobacillus*. The current result was consistent with previous studies identifying *Bacteroidetes* as the dominant phylum (>50% of sequences) and *Bacteroides* being the dominant genus in the cecum (about 40%) (Wei et al., 2013; Xiao et al., 2017). Previous study has shown that the probiotic mixture of *Lactobacillus pentosus* ITA23 and *Lactobacillus acidophilus* ITA44 increased beneficial bacteria population (including *Bifidobacteria, Lactobacillus*, and *Enterococcus*) and decreased *Escherichia coli* population in the cecal contents of broiler chickens (Faseleh Jahromi et al., 2016). *Lactobacillus acidophilus* D2/CSL (CECT 4529) supplementation provided in the drinking water improved beneficial microbes and functional genes in broiler crops and cecum (De Cesare et al., 2020), while feeding this strain in the diet improved the relative abundances of *Lachanospiraceae, Ruminococcus obeum, Clostridium clostridioforme, Roseburia intestinalis, Lachnospiraceae bacterium*, and *Coprococcus eutactus* in the cecal contents (De Cesare et al., 2017). Furthermore, *Bacillus* DFM could reduce the overgrowth of potential pathogenic bacteria, and promote the proliferation and production of beneficial bacteria and metabolites (Grant et al., 2018). Other studies also confirmed that *Bacillus subtilis* decreased the *Escherichia coli* and *Salmonella* populations in the cecum (Gao et al., 2017) and increased *Lactobacilli* numbers (Wu et al., 2011). Similarly, we found that dietary supplementation with heat-inactivated probiotics enriched the relative abundances of *Barnesiellaceae* (family), *Barnesiella* (genus), and *Lactobacillus aviarius* (species), while at the same time suppressing the relative abundances of *Lachnoclostridium, Peptococcus*, and *Lachnospiraceae* at d 42. At the same time, the pathogenic bacteria such as *Enterobacteriaceae* and *Escherichia coli* were enriched in NC group at d 21 when the broilers were fed the basal diets without antibiotic or compound probiotics. Moreover, the UPGMA analysis also indicated that the NC group at d 42 showed clear clustering from other groups, indicating the potential effectiveness of compound probiotics in regulating the gut microbiota composition of broilers. These differentially abundant bacteria might represent potential targets to improve animal growth performance via modulation of prebiotics and probiotics (Stanley et al., 2012).

It is evident that probiotics play important roles in modulating the composition, diversity, and function of the gut microbiota as well as maintaining the gut epithelium barrier function, and immune homeostasis (Azad et al., 2018). Previous study has shown that *Lactobacillus acidophilus* supplementation decreased the Shannon index of the ileal microbiota, and helped to restore the microbial community composition disrupted by *Clostridium perfringens* infection in male Arbor Acres broilers (Li et al., 2017). We showed that there was no significant difference in α-diversity of cecal microbiota in female yellow-feathered broilers, but dietary supplementation with heat-inactivated probiotics significantly increased the β-diversity index of cecal microbiota compared to the other two groups. Based on the predictive microbial function analysis of microbiota by PICRUSt, the metabolic pathways related to methane metabolism, transcription machinery, purine metabolism and protein export were enriched by heat-inactivated compound probiotics. Similar work on broilers revealed that live probiotic supplementation modulated the diversity and composition of ileal and cecal microbiota, and enriched pathways involved in carbohydrate metabolism whereas decreasing pathways of protein metabolism (Bortoluzzi et al., 2019).

However, few studies have demonstrated the associations of feed efficiency and plasma metabolites with altered gut microbiota composition in yellow-feathered broilers fed with compound probiotics of heat-killed *Bacillus subtilis* and *Lactobacillus acidophilus* BFI. We found that profiles of taxonomic composition in cecal microbiota were significantly associated with the overall feed efficiency and plasma metabolites in yellow-feathered broiler via Spearman's correlation analysis. Interestingly, the current study demonstrated a link between broiler overall feed efficiency with significant alterations of cecal microbiota species at genus level during the starter and grower phases, instead of the finisher phase. Specially, the relative abundance of *Subdoligranulum* was negatively correlated with overall FCR, which indicated that dietary supplementation with heat-inactivated compound probiotics could improve the feed efficiency potentially by increasing the abundance of *Subdoligranulum* as observed at d 21. Similar study conducted in neonatal calves has also indicated that the genera *Subdoligranulum, Blautia, Lactobacillus*, and *Bacteroides* were closely correlated with the health status (Jang et al., 2019). Furthermore, the relative abundance of *Lactobacillus aviarius* in cecal content was also enriched by heat-inactivated compound probiotics in this study. Consistently, the previous study has shown that the abundance of jejunal *Lactobacillus aviarius* abundance increased by dietary heat-inactivated compound probiotics was positively correlated with ADG of broilers via Pearson's correlation analysis (Chang et al., 2020). In fact, the gut microbiota can regulate the host health and disease by shaping the biochemical profile of the diet (Rowland et al., 2018). Here, we showed that the plasma metabolites including uric acid, creatinine, ALT, and cholesterol affected by dietary treatment of antibiotics or heat-inactivated compound probiotics, were closely related to the dynamic changes of broiler cecal microbiota composition. Thus, these results suggest that the beneficial effects of heat-inactivated compound probiotics in improvement of feed efficiency and reduction of plasma cholesterol and creatinine concentrations could be attributed to modulation of gut microbiota composition, diversity, and function in yellow-feathered broilers. However, the limitations of the present study failed to clarify the protective role and mechanism of heat-inactivated compound probiotics in regulating the gut microbiome, metabolism and health of broilers using specific pathogen challenge models, which needs further investigations.

## Conclusion

Collectively, the results of the present study suggested that modulation of gut microbiota through dietary heat-inactivated compound probiotics with *Bacillus subtilis* and *Lactobacillus acidophilus* BFI might contribute to the homeostasis and health of gut ecosystem, thus resulting in an improvement in feed efficiency and reduction of plasma cholesterol and creatinine concentrations in yellow-feathered broilers. These results would provide novel insights into the application of heat-inactivated compound probiotics, as a potential AGP replacement in poultry production, by potentially targeting the composition, functional capacity and diversity of gut microbiota. Future investigations should be carried out to elucidate the potential mechanism host-microbe interactions in metabolism and physiology for better performance and well-being of broilers.

## Data Availability Statement

The raw data supporting the conclusions of this article will be made available by the authors, without undue reservation.

## Ethics Statement

This animal study was reviewed and approved by Animal Care and Use Committee of Foshan University.

## Author Contributions

CZ, LG, and KH conceived the experiment, analyzed the data, and wrote the manuscript. FL and DT conducted the broiler trial. LG, KH, FL, and DT conducted laboratory analyses. HZ designed the whole trial and revised the manuscript. All authors read and approved the final version of the manuscript.

## Conflict of Interest

The authors declare that the research was conducted in the absence of any commercial or financial relationships that could be construed as a potential conflict of interest.
